# Exploring the impact of comorbid dementia on exacerbation occurrence in general practice patients with chronic obstructive pulmonary disease

**DOI:** 10.1177/14799731241280283

**Published:** 2024-09-17

**Authors:** Nicole OA de Kort, Erik WMA Bischoff, Michael Ricking, Tjard R Schermer

**Affiliations:** 1 Department of Primary and Community Care, 6034 Radboud University Medical Center, Nijmegen, The Netherlands; 2 Gelre Hospitals Apeldoorn and Zutphen, Zutphen, The Netherlands; 3 Department of General Practice, Erasmus MC University Medical Center, Rotterdam, The Netherlands; 4 Radboudumc Technology Centre Health Data, 6034 Radboud University Medical Center, Nijmegen, The Netherlands

**Keywords:** Chronic obstructive pulmonary disease, epidemiology, dementia, exacerbation, general practice, risk assessment

## Abstract

**Objective:**

Previous studies have shown an increased risk of dementia in patient with COPD, but whether comorbid dementia modifies the risk of exacerbations in patients with COPD is unknown. We explored exacerbation occurrence in patients with COPD with comorbid dementia and compared this to patients with COPD without comorbid dementia.

**Methods:**

We performed a retrospective cohort study based on medical record data from 88 Dutch general practices. Patients diagnosed with COPD and comorbid dementia (*n* = 244, index group) were matched 1:1 to patients with COPD without comorbid dementia (*n* = 244, controls). Exacerbations were assessed 1 year before and 1 year after the dementia diagnosis (or corresponding date in controls) and compared between index and control groups by calculating Rate Ratios (RRs).

**Results:**

Average number of COPD exacerbations after dementia diagnosis increased from 5 to 14 per 100 patient years in the index group (RR = 2.70, 95%CI 1.42-5.09; *p* = 0.02) and from 17 to 30 per 100 patient years in the control group (RR = 1.74, 1.19-2.54; *p* = 0.04). These RRs did not significantly differ between the index and control groups (RR ratio = 1.55, 0.74-3.25; *p* = 0.25).

**Discussion:**

We conclude that although the risk of exacerbation increased after patients with COPD were diagnosed with dementia, their change in exacerbation risk did not seem to differ from the change observed in patients with COPD without comorbid dementia. However, as our study was hypothesis-generating in nature, further investigations on the subject matter are needed.

## Introduction

Chronic obstructive pulmonary disease (COPD) and dementia are both chronic conditions that are mainly diagnosed in the older population. A typical feature of COPD is the occurrence of exacerbations: acute episodes in which symptoms and signs of COPD are increased.^1^ Approximately 20% of patients diagnosed with COPD in general practices suffer from one or more exacerbations per year.^2^

Dementia is characterized by two or more cognitive impairments that have an impact on daily life.^3^ Although dementia can also occur in younger adults, most people with dementia are diagnosed after the age of 65.^4^ In 2019, the number of people with dementia known to Dutch general practitioners was approximately 114,000, but the number of undiagnosed cases in the population is at least as high.^5^

Previous studies have shown that patients diagnosed with COPD are at higher risk of developing cognitive impairment, especially Alzheimer’s disease and vascular dementia.^6–12^ The pathophysiology behind the cognitive impairment is most likely a combination of long-term hypoxemia, systemic inflammation, and oxidative stress.^13^ COPD together with dementia can lead to increased risk of severe sepsis, acute respiratory failure, and death.^14^

Although the relationship between existing COPD and the risk of dementia seems clear, only two studies have been reported that have looked at associations between dementia and COPD exacerbations. Zarowitz *et al.* performed a study in nursing home residents with COPD who were cognitively and functionally impaired.^15^ Twenty-two percent experienced ≥2 exacerbations per year. Gupta *et al.* showed that patients who were admitted to hospital with COPD exacerbations and had coexisting dementia had worse outcomes in terms of length of hospital stay and in-hospital mortality.^16^ To our knowledge, no previous research has been reported on whether exacerbation occurrence changes after a patient with COPD is diagnosed with dementia.

Cognitive impairment in patients with COPD significantly increases the need for support in treatment adherence and effective self-management.^17^ Therefore, COPD exacerbation risk may change after dementia is diagnosed, since prevention and timely treatment of exacerbations depends – among other things – on treatment adherence and self-management. A first step in gaining knowledge on this and to inform primary and advance care practice would be to assess whether or not exacerbation risk changes when a patient with COPD is diagnosed with dementia. The aim of this hypothesis-generating study was therefore to compare occurrence of exacerbations in patients with COPD in the year before with the year after dementia was diagnosed. We also compared exacerbation occurrence in patients with COPD with dementia to a control group of patients with COPD without comorbid dementia.

## Methods

### Study design and data source

This retrospective matched cohort study was based on data extracted from the electronic patient journal systems of 88 general practices that are connected to the Department of Primary and Community Care of the Radboud university medical center (Radboudumc) in Nijmegen, the Netherlands for the years 2008 until 2022. These practices are all situated in the wider catchment area of the Radboudumc in the eastern part of the Netherlands (i.e. the provinces of Gelderland, North Brabant, and Limburg), in cities, urbanized areas, and middle-sized and smaller villages. Diagnostic coding in the journal systems used by the practices is based on the International Classification of Health Problems in Primary Care (ICPC). ICPC codes were used to select patients with COPD with or without comorbid dementia from the database (R95, ‘Emphysema, COPD’; P70, ‘Senile dementia’). Other data extracted were demographic characteristics, prescriptions of oral corticosteroids, and – if available – GOLD severity stage.^1^

All methods applied were carried out in accordance with the Dutch Code of Conduct for Health Research.^18^ Relevant protocols were approved by a medical ethics review board (METC Oost-Nederland). Obtaining informed consent from subjects and/or legal guardian(s) for publication of de-identified medical record information in a scientific report was waived by the METC Oost-Nederland (file number: 2020-6871).

### Study population

**Figure 1.**
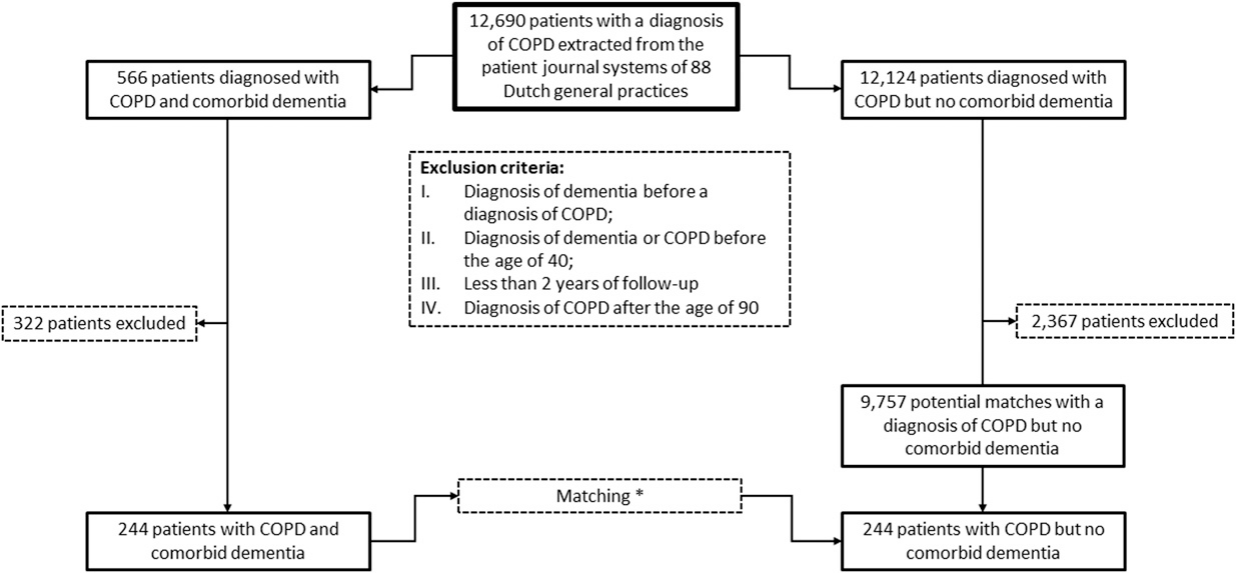
Flow diagram of selection and matching of patients with COPD with and without comorbid dementia. COPD: chronic obstructive pulmonary disease * 1:1 matching on sex, age at the time of dementia diagnosis in the index patient, and calendar year in which the dementia in the index patient was diagnosed. A control patient was selected from the same general practice as the index patient whenever possible. If several eligible controls for a particular index patient were present in the database, one of these potential controls was selected at random, and the others were dismissed as the matched control for this particular patient and placed back in the pool of potential controls to be available for matching to another index patient.

Patients diagnosed with COPD between January 2008 and December 2022 or who already had a diagnosis of COPD at the start of the data period were selected (Figure 1). Next, patients who were diagnosed with dementia before being diagnosed with COPD were excluded, as were patients who had a diagnosis of COPD or dementia before the age of 40, and patients who were diagnosed with COPD after the age of 90.

From the remaining patients with COPD, we selected those with at least 1 year observation time before, and 1 year observation time after being diagnosed with dementia as ‘index patients’. Thus, the total observation time for index patients was 2 years, with the date of the dementia diagnosis marking the middle of a patient’s observation period. Patients who had COPD without comorbid dementia were matched to index patients and also had at least 2 years observation time. To mark the middle of the observation period in a control patient, the date of the dementia diagnosis of the index patient to which the control patient was matched was used. This date was also used to select a recorded COPD GOLD severity stage in the past 2 years (if any) from an index or the matched control patient’s medical record.

### Matching procedure

Index patients were matched to control patients in a 1:1 ratio. To ensure the groups of index and control patients were comparable in terms of demographics and time frame, we used the following matching procedure. For each index patient identified in the database, one control patient with COPD but without comorbid dementia was selected. Matching criteria were: same sex, same age at the time of dementia diagnosis in the index patient (a maximum age difference of 2 years younger or older between the index patient and the matched control was allowed), and the calendar year in which the dementia in the index patient had been diagnosed. A control patient was selected from the same general practice as the index patient whenever possible. If several eligible controls for a particular index patient were present in the database, one of these potential controls was selected at random and the others were dismissed and placed back in the pool of potential controls for other index patients.

### Outcome

The outcome was the number of exacerbations per patient in the year before and the year after the diagnosis of dementia in the index patient or its matched control. Exacerbations were defined as prescriptions of oral corticosteroids (prednisolone (ATC H02AB06) or prednisone (ATC H02AB07)) with a minimum daily dose of 20 mg for 5 to 15 days. This is the treatment recommended for exacerbations in the Dutch College of General Practitioners COPD guideline.^19^ The average number of exacerbations per 100 patient-years was calculated for the year before and the year after the dementia diagnosis. We compared the occurrence of exacerbations before and after the date of the index patients’ dementia diagnosis and in the same time period for the matched controls.

### Statistical analyses

Analyses were performed using SPSS software (version 27, IBM SPSS Statistics, Feltham, Middlesex, UK). Statistical significance was defined as a two-sided *p*-value <0.05. We performed Poisson regression to calculate rate ratios (RRs) in the index and control groups separately to compare risk of exacerbation before and after the date of the index patient’s dementia diagnosis. Finally, we calculated the ratio of these two rate ratios (rate ratio ratio, RRR) to analyse whether or not there was a difference in the RR for COPD exacerbations in the years before and after the dementia diagnosis between the index and control groups. Post-hoc power was estimated for the RRs in the index and control groups using G*Power 3.1^20^ and for the RRR using the InteractionPoweR package in R Statistical Software (version R-4.4.1 for Windows).^21^

## Results

### Characteristics of the study population

Overall, data of 12,690 patients with a diagnosis of COPD were available from the general practice database (Figure 1).

After selection and exclusion, a total of 244 patients (51.6% males) with a diagnosis of COPD and comorbid dementia with a mean age of 78.1 (SD 8.0) years were included as index patients (Table 1).

**Table 1.** Baseline characteristics of the COPD patients with and without comorbid dementia.

	COPD with dementia (*N* = 244)	COPD without dementia (*N* = 244)
Mean age^a^ (SD), years	78.1 (8.0)	78.0 (7.8)
Median age^a^ (IQR), years	79.0 (10.0)	79.0 (10.0)
Age^a^ categories, *N*, (%)		
40-49 years	1 (0.4)	2 (0.8)
50-59 years	5 (2.0)	1 (0.4)
60-69 years	26 (10.7)	30 (12.3)
70-79 years	97 (39.8)	102 (41.8)
80-90 years	115 (47.1)	109 (44.7)
Sex, *N* (%)		
Male	126 (51.6)	126 (51.6)
Female	118 (48.4)	118 (48.8)
COPD GOLD stage, *N* (%)		
1	33 (13.5)	36 (14.8)
2	70 (28.7)	79 (32.4)
3	27 (11.1)	31 (12.7)
4	6 (2.5)	4 (1.6)
Unknown/missing	108 (44.3)	94 (38.5)
Chronic comorbid conditions^b^, *N* (%)		
Blindness or low vision	107 (43.9)	112 (45.9)
Peripheral vascular disease	74 (30.3)	66 (27.0)
Diabetes	52 (21.3)	53 (21.7)
Heart failure	42 (17.2)	51 (20.9)
Osteoporosis/osteopenia	33 (13.5)	35 (14.3)
Obesity	10 (4.1)	6 (2.5)
Depression	5 (2.0)	3 (1.2)
Lung cancer	0 (0)	1 (0.4)

^a^Age at the time of diagnosis of dementia and the fictitious date of dementia diagnosis in the control group of COPD patients without dementia, respectively.

^b^Selected chronic comorbid conditions as documented in patients’ general practice medical record and present during (part of) the 2-year observation period of a patient.

COPD: Chronic Obstructive Pulmonary Disease, GOLD: Global Initiative for Chronic Obstructive Lung Disease, IQR: interquartile range, SD: standard deviation.

A total of 9757 of patients with COPD without comorbid dementia were available as potential controls of which 244 served as matched controls (51.6% males, mean age 78.0 (SD 7.8) years).

In 44.3% of the index patients, the COPD GOLD stage could not be derived from the coded medical record data; in the controls this percentage was 38.5%. Among patients with known GOLD stage, GOLD-2 was the most common stage in both study groups (28.7% and 32.4% for index and control patients, respectively). Chronic comorbid conditions were common in both groups (Table 1).

### Number of exacerbations

Table 2 shows the numbers of COPD exacerbations per patient before and after the date of diagnosis of dementia (or the corresponding date for controls).

**Table 2.** Number of COPD exacerbations per patient and average number of exacerbations in 100 patient years in the COPD patients with and without comorbid dementia.

	Exacerbations	
	BEFORE date of dementia diagnosis	AFTER (fictitious^a^) date of dementia diagnosis
COPD with dementia (*N* = 244)		
Average number of exacerbations per 100 patient years (SD)	5 (1.5)	14 (2.4)
Category of number of exacerbations per year, *N* (%)		
0	235 (96.3)	224 (91.8)
1	6 (2.4)	10 (4.1)
2	2 (0.8)	6 (2.5)
3	1 (0.4)	3 (1.2)
4	0 (0.0)	1 (0.4)
5	0 (0.0)	0 (0.0)
COPD without dementia (*N* = 244)		
Average number of exacerbations per 100 patient years (SD)	17 (2.7)	30 (3.5)
Category of number of exacerbations per year, *N* (%)		
0	220 (90.2)	201 (82.4)
1	13 (5.3)	25 (10.2)
2	5 (2.0)	9 (3.6)
3	5 (2.0)	7 (2.9)
4	1 (0.4)	1 (0.4)
5	0 (0.0)	1 (0.4)

COPD: Chronic Obstructive Pulmonary Disease.

^a^date on which the index COPD patient to which a control patient was matched was diagnosed with dementia. This date marked the cut-point between the first and second observation year in the control patient.

Among the patients with comorbid dementia, 3.7% had ≥1 COPD exacerbations in the year before the dementia diagnosis, whereas 8.2% had ≥1 COPD exacerbations in the year after the dementia diagnosis. The corresponding proportions among the matched controls were 9.8% and 17.6%, respectively.

In the group with comorbid dementia, the average number of exacerbations in the year before the dementia diagnosis was 5 (SD 1.5) per 100 patient-years and after dementia diagnosis 14 (SD 2.4) per 100 patient years (Table 2). This amounts to a 169% increase in the average number of exacerbations, corresponding to a RR of 2.70 (95%CI 1.42 to 5.09; *p* = 0.02; post-hoc power 0.87, Table 3).

**Table 3.** Comparison between annual exacerbation rate ratios in the COPD patients with comorbid dementia and the control group of COPD patients without comorbid dementia. The rate ratios of the ‘COPD with dementia’ and ‘COPD without dementia’ groups show the comparison between the average number of exacerbations in the year prior to the dementia diagnosis relative to the number of exacerbations in the year after the dementia diagnosis. The rate ratio of the group ‘Dementia versus control group’ shows the ratio of the rate ratios (‘rate ratio ratio’) of the groups with comorbid dementia and the group without comorbid dementia. Statistically significant *p*-values (i.e., *p* < 0.05) are printed **bold**.

	Rate Ratio	95% CI	*p-*value^a^	Post-HOC power estimate
COPD with dementia (N = 244)	2.70	1.42, 5.09	**0.02**	0.87
COPD without dementia (N = 244)	1.74	1.19, 2.54	**0.04**	0.82
	Rate Ratio Ratio	95%CI	*p*-value^a^	
COPD with dementia relative to copd without dementia (N = 488)	1.55	0.74, 3.25	0.25	0.16

CI: confidence interval; COPD: chronic obstructive pulmonary disease.

^a^from Poisson regression model.

Controls showed an average number of 17 (SD 2.7) exacerbations per 100 patient-years in the year before and 30 (SD 3.5) exacerbations per 100 patient-years in the follow-up year (Table 2). This amounts to a 74% increase in the average number of exacerbations, corresponding to a RR of 1.74 (95%CI 1.19 to 2.54; *p* = 0.04; post-hoc power 0.82, Table 3). Patients with comorbid dementia did not show a statistically significant difference for the RR of COPD exacerbations relative to the RR in patients without comorbid dementia (RRR of 1.55 (95%CI 0.74 to 3.25; *p* = 0.25; post-hoc power 0.16, see Table 3).

## Discussion

This study seems to be the first to assess occurrence of exacerbations in patients with COPD in the period before and after dementia is diagnosed and to compare this with patients with COPD without comorbid dementia. Our observations showed an increase in exacerbations in patients with COPD with comorbid dementia over time, but this was also seen in the control group. In the year before dementia was diagnosed in the index patients the exacerbation rate was lower in patients with comorbid dementia than in the control patients. In the year after the dementia diagnosis, the exacerbation rate remained higher in the control patients. The change in the rate ratio for exacerbation after being diagnosed with dementia was 1.55 times the rate ratio in control patients, which was not statistically significant.

One possible explanation for our findings might be the underreporting of COPD exacerbations in general and especially in patients with (yet) undiagnosed dementia. Langsetmo *et al.* showed that patients with COPD report less than a third of all exacerbations.^22^ Although no research has been reported on the relationship between dementia and underreporting of COPD exacerbations specifically, it is quite conceivable that the patients who were diagnosed with dementia were less capable of recognizing and reporting symptoms and signs of COPD exacerbations. It takes about 2 years to recognize the first symptoms of dementia and about 3 years before a dementia diagnosis is formally established.^23^ This could explain the lower number of exacerbations in the first year of observation in the patients with dementia in our study since these patients may already have had (cognitive symptoms of) dementia in that period. The increasing number of exacerbations we observed in this group may be explained by an increased exacerbation risk due to worsening of the COPD, although this may also be true for the controls. Another explanation may be that patients received more care, attention, and support after their dementia was diagnosed. Changed involvement of (formal and informal) caregivers who may be more capable in noticing exacerbations than the patient him/herself may have reduced underreporting of exacerbations.^22^ These caregivers may also have ensured that COPD-related treatment compliance improved and that non-pharmacological interventions (e.g., influenza vaccination) were better adhered to. Although beforehand we hypothesized that insufficient self-care and therapy compliance in the period before dementia diagnosis would show higher numbers of exacerbations in this period, this may have been compensated through more explicit attention given by caregivers.

The main strength of our study is the large general practice database from which we could select patients with COPD with comorbid dementia as well as matched COPD controls without dementia. We have used this data source in several previous studies.^24–26^ ICPC coding was used in all participating general practices as a standardized way of recording diagnoses of COPD and dementia.

A clear limitation of this study is the fact that we were limited to the use of information that was recorded in patients’ medical records during routine care provision. Consequently, the data were not always complete, which may have led to confounding (for instance, we could not match on and/or adjust the analysis for differences in smoking history or smoking status between the two groups due to incomplete data regarding smoking in the medical records). The same is true for other relevant lifestyle (e.g., treatment incompliance) and demographic (e.g., socioeconomic status) characteristics that may be related to exacerbation risk.

Another limitation is the fact that we do not have data regarding the role of formal caregivers (e.g., community nurses, home care staff) nor informal caregivers (e.g., spouses, neighbours, volunteers) in noticing and reporting (imminent) exacerbations. Such information is not recorded in general practice medical records and it would require other data sources and/or a qualitative research design to assess and comprehend this.

The main strength of the matching procedure we applied is that patients in the two study groups were comparable in terms of sex, age, calendar period from which their data were used, and general practice in which they were registered. Thus, the calculated RRs and RRR are well corrected for these factors. The main limitation is that as a consequence of the choice to match for these four factors, we could not use data from a substantial number of potential matches (i.e., 9757 - 244) because despite the apparent abundance of potential controls, a 1:1 matching rate was the highest achievable rate before the pool of potential controls ran out of matches.

We used the predniso(lo)ne prescription-based definition of exacerbation previously used by Westerik *et al.*^25^ and reflects the recommendation for COPD exacerbation management in the Dutch College of General Practitioners guideline,^19^ which is similar to recommendations in international COPD guidelines.^1,27,28^ This means that exacerbations that did not require predniso(lo)ne treatment were not included in our analysis. Although this is true for both the index and the control patients, this may have distorted the results. Despite the significant number of patients with COPD in our study (244 with and 244 without comorbid dementia), the vast majority did not have documented exacerbations at all, which limits the statistical power of our study. Because of this and because of the aforementioned lack of some relevant data we did not correct the analysis for comorbid conditions^25^ or other factors that are associated with an increased risk of COPD exacerbations. Also, we did not look at (changes in) prescribed respiratory medication (i.e., inhaled corticosteroids) that may have affected exacerbation risk.

Zarowitz *et al.* found a higher rate of COPD exacerbations: 22% of the patients experienced at least two COPD exacerbations during a 12-months follow up period.^15^ However, this study was performed in nursing homes in patients with cognitive impairment, whereas our data were derived from general practices. In nursing homes, with many health professionals closely involved in daily care provision, it is likely that COPD exacerbations are recognized more often than in a general practice setting. Another explanation for the difference in exacerbation rate between the two studies could be that patients with COPD who reside in nursing homes suffer from more severe COPD.

Dementia and COPD are both slowly developing conditions, and the point at which either diagnosis is finally made is largely determined by the family/social context, which is arguably a major influence on when healthcare help is sought and a diagnosis confirmed. Thus, the point of diagnosis is not a specific point in time so much as a context-determined threshold that has been breached – most likely when the patient is so frail or the social support so exhausted that an increase in dependence on the healthcare services is inevitable. The increase in exacerbations in both study groups might therefore be evidence of increasing frailty, but unfortunately structured assessments of frailty or indicators of frailty such as referrals to social services are not available in our database.

Since presumably exacerbations may go unnoticed more often in patients with COPD in an early stage of dementia, specific attention with regard to supervision of COPD treatment compliance and support of self-care may be appropriate in these patients. This applies not only to the time after a formal diagnosis of dementia has been made, but also beforehand when a GP suspects cognitive decline.

In conclusion, this study appears to be the first attempt to assess the impact of comorbid dementia on the risk of exacerbations in patients with COPD. Although exacerbation occurrence increased after patients with COPD were diagnosed with dementia, their change in exacerbation risk did not seem to differ from the change observed in patients with COPD without comorbid dementia. Because our study was hypothesis-generating in nature the results should be interpreted with caution and translation of the conclusions to clinical practice avoided. Further research is needed first to elucidate the impact of (the cognitive impairments preceding a diagnosis of) dementia and frailty on patients’ with COPD respiratory health, not just in terms of exacerbations but also for other relevant health outcomes and for the way respiratory care is provided.

## Data Availability

The data are not made publicly accessible because variable names, labels, and codebooks are all in the Dutch language. A de-identified dataset can be requested from the corresponding author, in which case relevant variables and labels will be translated to English.*
